# Preschoolers’ temperament and social functioning in novel and routine contexts

**DOI:** 10.3389/fpsyg.2022.975110

**Published:** 2022-12-22

**Authors:** Helena Shoplik Vaughan, Hedwig Teglasi

**Affiliations:** Department of Counseling, Higher Education, and Special Education, University of Maryland, College Park, MD, United States

**Keywords:** temperament, negative emotion, positive emotion, effortful control, context, children

## Abstract

**Introduction:**

The centrality of social competence to children’s short and long-term well-being has sparked interest in the factors that contribute to its development, including temperament, a set of biologically based dispositions. A large body of work documents two types of temperamental dispositions associated with young children’s social functioning: reactivity and regulation. There is consensus about the detrimental effects of negative reactive tendencies, called negative affective reactivity (NA), and about the beneficial effects of regulatory tendencies, called effortful control (EC), on social functioning. Another reactive component of temperament, Extraversion/Surgency (E/S) is less consistent in its relation with social functioning. Although NA is exacerbated by lack of familiarity, its contribution to social functioning in novel and routine contexts has not been systematically addressed.

**Methods:**

To test this study’s hypotheses, we devised a structured interview of adaptive responsiveness in context (ARC) which was completed by parents of preschoolers along with a comprehensive temperament questionnaire. Additionally, children completed an individually administered task measuring emotion-situation knowledge (*N* = 92) and their teachers completed a standard social competence questionnaire.

**Results and Discussion:**

A path analysis that controlled for variance shared across contexts and temperamental traits showed that NA was the only unique predictor of social functioning in the Novel context, that EC was the only unique predictor of social functioning in the Routine context and that E/S was not a unique predictor of social functioning in either context. Bivariate analyses, conducted without controlling for context overlap, showed all reactive emotional traits (subsumed within NA and E/S) to correlate exclusively with ARC in the Novel contexts. However, regulatory traits showed a mixed pattern. Inhibitory Control correlated with ARC in both contexts but more highly in the Routine context, and Perceptual Sensitivity correlated with ARC in the Novel context.

## Introduction

The centrality of young children’s social competence to their well-being, both in the short-and long-terms has sparked interest in research to understand factors contributing to effective social functioning, including temperament. Conceptualized as a set of biologically based dispositional traits, temperament is thought to remain relatively stable over time and situation, though amenable to influence by experience (see Rothbart and Bates, 2006). A large body of work documents relations between temperament and children’s social competence (SC) as rated by adult informants in various settings (i.e., home, school, or peer group; Denham et al., 2001; Denham, 2006). However, the measures of SC are context free.

Contexts vary in their requirements for children to adjust to novel or intense stimuli, to size up subtle cues, or to restrain their behavior. An important determinant of social competence is the appropriateness of the child’s behavior in the context (Rose-Krasnor, 1997) and, it is not unusual for the same child to be rated as socially competent in one setting but not another (Teglasi et al., 2017). In this vein, discrepancies between adult informants, particularly those observing children in different settings, such as home and school, are commonly found, and increasingly attributed to differences in those contexts (Dirks et al., 2012; Teglasi et al., 2015). However, treating these settings broadly as proxies for context leaves gaps in our understanding of what aspects of situations may be challenging for children with particular temperamental dispositions. Temperamental individuality influences what children notice in their surroundings, how they react, and how they elicit responses from others, thereby influencing their transactions, hence the ratings of informants, in context-specific ways.

### Temperament and social functioning

Rothbart proposed two overarching processes of reactivity and regulation that subsume various temperament traits (Rothbart, 1989). Reactivity includes affective, motoric, and sensory responses to internal and external stimuli, and regulation includes attentional and inhibitory responses to modulate reactivity. This framework is supported by the three-factor structure characterizing the Rothbart family of questionnaires (see Rothbart et al., 2001; Teglasi et al., 2015), which includes one regulatory component, Effortful Control (EC) and two reactive components, Negative Affectivity (NA) and Extraversion/Surgency (E/S).

The Negative Affectivity (NA) component of temperament encompasses dispositional tendencies to experience and to remain in unpleasant emotional states (Anger, Fear, Sadness, Discomfort and Falling Reactivity). Inverse associations between NA and children’s social competence (SC) are consistently documented (for a review, see Fabes et al., 2008; Eggum-Wilkens et al., 2016), even among preschoolers (see Denham et al., 2001; Kolak et al., 2013).

Effortful Control (EC), the regulatory component of temperament, includes four subscales that, when composited, evidence consistent positive relations with socially effective behaviors (Eggum-Wilkens et al., 2016). In aggregate, EC is thought to play a role in moderating the intensity of NA (e.g., Denham et al., 2001; Gartstein et al., 2012) and in mitigating the adverse impact of NA on social functioning (Eisenberg et al., 2004; Acar et al., 2015; Eggum-Wilkens et al., 2016). Overall, EC is thought to enable the individual to refrain from a ‘ready’ response and to resist distraction in order to maintain attention on a task (Rothbart and Putnam, 2002). When EC subscales are disaggregated, positive associations with SC are consistently demonstrated with Attentional Focus and Inhibitory Control (Eisenberg et al., 2016). The two other subscales, Perceptual Sensitivity and Low Intensity Pleasure, are relatively understudied in relation to SC.

The Extraversion/Surgency (E/S) component of temperament encompasses tendencies to experience positive emotions (Smiling/laughter) and to engage in behaviors described as exuberant (Activity, Approach, Low Shyness, Impulsivity). Although the relation between Extraversion/Surgency (E/S) and social behavior has been studied, the majority of the research has focused on Negative Affectivity (NA) and Effortful Control (EC). Overall, E/S appears less predictive than NA of children’s social outcomes (Sanson et al., 2004; Slagt et al., 2016). When the E/S subscales are disaggregated, there is evidence that, in young children, Smiling is linked with higher social competence and that Shyness is associated with lower social competence (e.g., Teglasi et al., 2015).

### Context-specificity

Pre-schoolers often encounter new experiences that ordinarily become routine over time and face unanticipated departures from routines or expectations. Uncertainties inherent in unfamiliar or unexpectedly changing situations increase feelings of fear or discomfort (Morriss et al., 2022), particularly in individuals who are prone to higher NA reactivity, and may also heighten other negative emotions such as sadness or anger (Bar-Anan et al., 2009). Hence, the disruptive effects of NA on functioning are particularly apparent in the face of novelty or departures from expectations (Kagan, 1997). These disruptive effects extend to the influence of NA on how children process information in their surroundings (e.g., Pérez-Edgar et al., 2011). When children are gripped by intense negative emotions, concerns about their emotional states may interfere with the strategic deployment of EC to attune information processing and behavior to the requirements of the context (e.g., Taylor et al., 2014; Bonmassar et al., 2020). Children with higher NA may direct their EC toward processing the immediate cues in the surroundings in an attempt to tamp down affective reactivity, but detracting from the pursuit of social goals. Hence, when facing novel or unexpectedly changing conditions, children with higher NA may show behaviors that appear less flexible and therefore less likely to be rated as socially competent.

As the context becomes more familiar, pre-existing understandings contribute to changes in emotions elicited and put children in a better position to direct their EC toward purposeful actions that are responsive to the context and are observable to others. In the novel context, higher EC may not compensate directly for the adverse effects of NA on social functioning, but may work indirectly by enabling children to gain social–emotional understandings that reduce uncertainty with increasing familiarity (e.g., Verron and Teglasi, 2018). Children who are less able to maintain their negative emotional reactivity within a tolerable range may need more time to gain or to access the social emotional understandings that underlie effective social behaviors. For these reasons, the independent contribution of EC to social functioning, documented in the literature, may be undermined in the novel context, whereas the independent contribution of NA, documented in the literature, may be mitigated in the routine context.

As a reactive component of temperament, E/S seems particularly salient to functioning in novel contexts. Even so, the extent of its contribution is unclear when the effect of NA or EC is controlled. Research and theory suggests that both high and low levels of E/S detract from behavior deemed socially competent, albeit in different ways. Children who are low in surgency tend to be apprehensive when faced with new experiences, hence to be cautious, inhibited, or avoidant in such situations (Hipson and Séguin, 2015). However, given substantial evidence that NA contributes to socially inhibited/avoidant behaviors in the face of novelty, we expect that controlling for NA would mitigate the unique contribution of E/S to adaptive functioning in novel contexts. On the other hand, children who are high in surgency tend to be exuberant (highly active in exploring their surroundings) in their responses to novelty, but may sometimes behave in ways that disregard rules and standards for appropriate behaviors (Sallquist et al., 2009; Dollar and Stifter, 2012). For these reasons, we do not expect the E/S composite to uniquely predict ARC in the Novel context. However, we anticipate that at least two of its subscales, Shyness and Smiling, will correlate with ARC in the Novel context.

### Current study

Parents and educators are well-positioned to observe young children’s social functioning in a wide range of situations, but questionnaires to assess social competence do not incorporate context. Although associations of NA and EC with social functioning are extensively documented, little is known about the independent contribution of each to functioning in novel and routine contexts. To pursue the aim of this study, we devised a structured parent-interview to measure children’s adaptive responsiveness in novel and routine social contexts (Adaptive Responsiveness in Context; ARC).

The context sets the requirements for effective responding to which temperamental reactivity and regulation may be differentially salient. At about age four, stable individual differences begin to emerge in both regulation and reactivity (Kochanska et al., 2000). For preschoolers, the transformation of an unfamiliar encounter to a familiar one is commonplace. With increasing experience, some aspects of new situations may become predictable, whereas some aspects of familiar situations remain uncertain. Features of unfamiliar contexts that increase predictability (i.e., structure, rules, clear expectations) may reduce uncertainty whereas certain features of familiar contexts (i.e., ambiguities of peer interactions) may heighten uncertainty. For these reasons, preschoolers’ functioning in novel and routine contexts are likely distinct, but also related.

### Hypotheses and data analyses

#### Validation of the adaptive responsiveness in context

To develop the ARC as a measure of adaptive responsiveness in context, we conducted pilot interviews with parents of preschoolers and generated 18 items to measure aspects of functioning that are salient in contexts that are familiar/routine (e.g., understanding implicit rules, following clear instructions) and in contexts that include elements of novelty or ambiguity (e.g., unexpected change, peer interactions). We investigated the properties of this measure to ascertain its validity as a tool for this study, Principal components analyses (including examination of the scree plot and parallel analyses; Bryant and Yarnold, 1995) supported a two-factor solution distinguishing between adaptive responses in Novel and Routine social contexts. Subsequently, we examined the feasibility of using Novel and Routine contexts as subscales of the ARC. We hypothesized that the scales would be internally consistent and that they would correlate differentially with relevant variables. Since preschool teachers emphasize routines, we expected that Social Competence, which captures conventional prosocial behaviors, would correlate with ARC in the Routine but not in the Novel context and that Internalizing behaviors, which are rooted in negative affectivity, would correlate with ARC in the Novel, but not Routine context. In view of the importance of social cognitions as shaping children’s observable behaviors, we expected that scores on the ARC would correlate with children’s emotion-situation knowledge, measured with the Emotion Comprehension Test (ECT; see Verron and Teglasi, 2018). However, since the ECT samples conventional situations, we expected correlations to reach significance in the Routine, but not in the Novel, context.

#### Relations of temperament with social functioning

Studies relating temperament with social functioning often aggregate scales subsumed within the three broader temperament factors. We relied on these factors as well to test our central hypothesis. However, we also examined the bivariate relations between each of the 15 subscales of the Child Behavior Questionnaire (CBQ; Rothbart et al., 2001; Putnam and Rothbart, 2006) with ARC in the Novel and Routine contexts. Assuming that the requirements of novel and routine contexts are distinct but somewhat overlapping, we tested an exploratory path model that includes AR in Novel and in Routine contexts as the outcome variables and temperament traits as predictor variables. The inclusion of both contexts in the model was intended to control for any existing overlaps between them to highlight the unique, context-specific relations of reactive and regulatory temperamental dispositions with ARC.

We hypothesized that effortful control (EC) is a unique predictor of adaptive social responding (AR) in routine contexts and that negative affectivity (NA) is a unique predictor of AR in novel contexts. Although we expected reactive tendencies, including those subsumed within the E/S composite to be more salient to adaptive responding in Novel than in Routine contexts, we did not expect that the E/S composite would make a unique contribution beyond NA and EC. By virtue of controlling for other variables, the partial correlations in the path model are distinct from the bivariate relations of each temperament subscale with ARC. Missing data were addressed by using full information maximum likelihood (FIML) which is the default setting in MPLUS. Using FIML assumes that data are missing at random and creates a covariance matrix taking into account the information in both complete and incomplete cases.

## Materials and methods

### Participants

Participants included children between 3 and 6 years of age (*N* = 92), enrolled in a private preschool on the campus of a large, public university in the Mid-Atlantic region of the United States as well as their parents and teachers. This pre-school is supportive of research conducted on campus and has established procedures for the conduct of studies. The sample comprised 54% European Americans, 13% African Americans, 13% Asian Americans, and 17% “other.” The mean age of participating children was 55.97 months (SD = 9.97), and girls made up 50 percent of the sample. Based on parental reports about their current employment, no one indicated having a position that would require only a high school level education, 24.6 percent reported having positions that require at least a 4 year college degree, and 29.6 percent reported positions that require a professional or graduate level degree. About half the participants, 45.8 percent, chose not to report this information.

### Procedures

In accord with procedures established by the IRB, parents gave informed consent and children gave their assent when picked up from class. No child declined to participate. At the time of data analysis, participants are identifiable only by number. To recruit participants, researchers made a presentation during back to school night. Subsequently, informed consent forms were sent home with students in six classrooms. Parents who consented to the study were provided with packets that included questionnaires with instructions for how to return completed forms to researchers. Packets were distributed to teachers of children in six classrooms whose parents gave consent. Trained graduate students conducted interviews, either in person or on the phone, with parents, primarily mothers and also administered the ECT individually to each participating child.

### Measures

#### Children’s behavior questionnaire

The CBQ was designed as a highly differentiated and comprehensive measure of temperament based on the conceptualization of temperament as individual differences in reactivity and regulation (Rothbart et al., 2001). The Short Form of the CBQ (Putnam and Rothbart, 2006) includes 94 items rated on seven-point Likert rating scales with response options ranging from 1 (extremely untrue of your child) to 7 (extremely true of your child). Parents are also provided with a Not Applicable response option. Each of the 15 subscales demonstrated adequate internal consistency in the current study, including Activity Level (*α* = 0.69), Anger/Frustration (*α* = 0.80), Approach/Positive Anticipation (*α* = 0.68), Attentional Control (*α* = 0.78), Discomfort (*α* = 0.86), Falling Reactivity/Soothability (*α* = 0.79), Fear (*α* = 0.74), High Intensity Pleasure (*α* = 0.74), Impulsivity (*α* = 0.73), Inhibitory Control (*α* = 0.65), Low Intensity Pleasure (*α* = 0.70), Perceptual Sensitivity (*α* = 0.76), Sadness (*α* = 0.65), Smiling and Laughter (*α* = 0.61), and Shyness (*α* = 0.86). There is considerable consensus that these subscales fall into three higher order factors, the regulatory factor of Effortful Control, and the reactive factors Negative Affectivity and Extraversion/Surgency (Rothbart et al., 2001). Composite scores were created by averaging applicable item scores.

#### Emotion comprehension test

The ECT (see Verron and Teglasi, 2018) was designed as an adaptation of the Affect Knowledge Test and the Assessment of Children’s Emotion Skills (ACES) for preschoolers. The ECT consists of three parts but only one is used in the current study, the Emotion-Situation Knowledge task (ESK), comprising 15 vignettes that are read and acted out by the researcher using two puppets. Participants are asked what the person in the story might be feeling. One example of a vignette is the following: “Green let Red play with Green’s favorite toy. Red plays with the toy and then it breaks. Do you think Green feels happy, sad, mad, scared, or no feeling?” To score the measure, two points are awarded for the correct answer and correct valence of emotion, one point is awarded for the incorrect answer but correct valence of emotion, and 0 points for answers with an incorrect emotion and incorrect valence. For 3 of the 15 scenarios, including the one described above, both sad and mad were given full credit. The “correctness” of the responses was determined in two ways: (a) consensus among the panel of 5 psychology doctoral students and a faculty member; and (b) a pilot test with preschoolers who were also asked to tell why the puppet would feel that way. With respect to three vignettes, the authors determined that both sad and mad would be appropriate given the discussion with the panel and children’s interpretation (e.g., sad when toy broke by accident or mad when on purpose). This rationale was supported by the finding of a bimodal distribution for these scenarios with sad and mad chosen most often and with relatively equal frequency.

The total score was calculated by adding the points awarded to individual items (*M* = 35.12, SD = 5.99). No significant differences were found between girls’ and boys’ scores. Internal consistency was adequate (*α* = 0.80). On average, administration time for the ECT was roughly 30 min.

#### Social competence and behavior evaluation

The SCBE (Preschool Edition; LaFreniere and Dumas, 1996; Anthony et al., 2005) is a teacher report questionnaire that describes a child’s functioning within a preschool classroom. Its 80 items measure overall emotional expression, social interactions with peers, and interactions with teachers on a 6-point scale from 1 (almost never occurs) to 6 (almost always occurs). Content of items ranges from asking about negotiating solutions to conflicts with other children to asking about bullying behaviors.

Teachers completed the entire SCBE questionnaire, but to test our hypotheses we utilized two of the three scales, the Internalizing scale (which measures depressive, anxious, or isolative behaviors) and the Social Competence scale (which measures prosocial behaviors). Studies investigating the psychometric properties of the SCBE have found the internal consistencies of these subscales to be high, with the internal consistency of all 80 items at *α* = 0.95 and the Externalizing scale (*α* = 0.94), Internalizing scale (*α* = 0.86) and the Social Competence scale (*α* = 0.94) each having high internal consistencies as well (Anthony et al., 2005).

#### Adaptive responsiveness to context scale

Parents are well-positioned to observe children’s social functioning in a wide range of encounters, but parent-report questionnaires to assess preschoolers’ social competence do not incorporate context. To pursue this study’s aims, we devised the Adaptive Responsiveness to Context (ARC) Scale to measure aspects of functioning that are salient in contexts that are familiar/routine (e.g., understanding implicit rules, following clear instructions) and unfamiliar, including elements that are novel, unexpected, or changing (e.g., emotional expression when faced with change, peer interactions). Based on pilot interviews with parents of preschoolers, we generated 18 items that seemed to differ in their implications for functioning in routine and novel contexts. This conceptualization was supported by the emergence of two internally consistent factors demonstrating theoretically meaningful relations with external correlates (see Results section). An example of a question that loaded on the Novel factor is “How does the child react to the postponement of a planned positive trip?” with response options on a Likert scale from “1. Distress, disappointment, and insistence on sticking with the plan” to “5. Takes it in stride, accepts it easily.”

Adaptive responses, including those captured by items on the ARC, are inherently self-regulated. Hence, it is reasonable to examine conceptual and item overlaps between the ARC and EC, the regulatory component of temperament. Three of the four EC scales involve basic regulatory processes that support, but do not directly capture, socially adaptive responses (*attentional focus*, *perceptual sensitivity*, and *enjoyment of low intensity* stimuli). One of the scales (*inhibitory control*), which emphasizes the capacity to suppress inappropriate behavior, does get at regulation in ways that directly relate to adaptive social responses, but does so more narrowly than does the ARC. This difference in conceptual scope is mirrored at the item level, with EC items describing specific behaviors (e.g., *can wait before entering a new activity when asked to do so; can easily stop an activity when told no*) and ARC items describing broad tendencies (e.g., *follows implicit rules*).

## Results

### The adaptive responsiveness to context scale

The ARC was developed in the current study as a way to measure adaptive responding (AR) to familiar and unfamiliar contexts. As part of its development, we conducted a factor analysis, including parallel analysis (see Table 1). We also examined its relations with teacher reported social competence and internalizing behavior (SCBE), and with child performance on the emotion-situation-knowledge (ECT).

**Table 1:** Factor loadings for exploratory factor analysis with varimax rotation for ARC scale.

ARC item	Routine	Novel
Item 98: Organized/planned vs. haphazard/unplanned behavior	**0.758**	
Item 110: Understands rules vs. requires external limits	**0.735**	
Item 97: Anticipates others’ reactions vs. surprised by reactions of others	**0.721**	
Item 109: Follows rules and standards vs. immediate wish	**0.708**	
Item 101: Following implicit rules without being told	**0.645**	
Item 100: Following clear instructions	**0.643**	
Item 107: Handles routine demands at home	**0.521**	0.368
Item 111: Acting without prior thought vs. careful forethought	**0.477**	−0.393
Item 99: Size up demands of new task or change in routine	**0.456**	
Item 112: Likelihood of planning ahead	**0.437**	
Item 106: Handles routine demands at school	**0.424**	0.365
Item 104: Responds to changes in situation		**0.745**
Item 103: Reacts to departure from expectations		**0.727**
Item 102: Responds to postponement of positive events		**0.602**
Item 47: Appropriateness of negative emotions		**0.501**
Item 46: Appropriateness of positive emotions		**0.479**
Item 105: Reacts to unexpectedly difficult activity		**0.365**
Item 108: Handles routine demands of peers		**0.359**
Eigenvalue	4.824	2.330
Cumulative percent of variance	26.800	39.743
Internal consistency	0.831	0.688 (*0.716*)
Number of items in subscale	11	7

Extraction method = principal components analysis; rotation method = Varimax with Kaiser Normalization (rotation converged in three iterations). Highest factor loadings are indicated in bold. Internal consistency estimates in italics were adjusted to a scale length of eight items using the Spearman-Brown Prophecy Formula; ARC = Adaptive Responsiveness in Context Scale.

#### Factor analysis

PCAs of the ARC showed two internally consistent factors, the Novel (*α* = 0.69) and the Routine (*α* = 0.83), which included 7 and 11 respective items (see Table 1). When internal consistency for the Novel subscale was adjusted to account for a low number of items with the Spearman-Brown Prophecy Formula for an 8-item scale, it was found to be acceptable at *α* = 0.71. AR across the two categories of contexts were moderately correlated, hence distinct but related (*r* = 0.369, *p* < 0.001).

As anticipated, there was a significant difference (*t* = 4.65, *p* < 0.001, df = 178) between mean ratings for the Routine (3.61; SD = 0.56) and for the Novel subscales (3.31; SD = 0.55) with Routine being higher. The mean parent rating on the Novel subscale was 3.24 (SD = 0.5439) for boys and 3.37 (SD =0.557) for girls. For the Routine subscale, the mean score for boys was 3.56 (SD = 0.599) and for girls the mean score was 3.65 (SD =0.509). Parent ratings were not significantly different between boys and girls for either subscale.

Scores from the Routine Adaptive Responsiveness subscale correlated significantly with age in months (*r* = −0.22, *p* < 0.05). Hence, subsequent analyses with this variable controlled for age.

#### External correlates

Bivariate correlational analyses showed context-specific patterns in keeping with expectations (see Table 2). In conducting these correlational analyses with teacher rated SCBE scales, we did not control for rater effects, which, after controlling for age in months, were small (ranging from 0 to 0.04) and non-significant.

**Table 2:** Correlations between adaptive responsiveness in context subscales, ECT subscales, and SCBE subscales.

Measures	Routine	Novel
Situations (ECT)	0.434**	0.162
Social competence scaled score (SCBE)	0.273*	0.045
Externalizing problems (SCBE)	0.157	0.113
Internalizing problems (SCBE)	0.188	0.260*

**p < 0.01 and *p < 0.05.

As anticipated, children’s ARC scores in Routine contexts correlated with ESK scores as measured by the ECT (*r* = 0.43, *p* < 0.01), which presents commonly occurring scenarios. A Fisher’s *z* test showed that the correlation between the ESK and the Routine subscale was significantly different from the correlation between ESK and the Novel subscale scores (*r* = 0.16, *z* = 2.21, *p* < 0.05).

Social competence scores correlated with ARC in the Routine context (*r* = 0.27, *p* < 0.05) but not with ARC in the Novel context. A Fisher’s *z* test indicated that this difference was significant (*z* = 1.57, *p* < 0.05). This pattern was as expected, given that pre-school teachers emphasize classroom routines. Also in line with expectations, scores on the Internalizing Problems subscale of the SCBE, which captures affective dysregulation, correlated with ARC in the Novel (*r* = 0.26, *p* < 0.05) but not in the Routine context. However, the difference between these correlations was not statistically significant.

Taken together, these correlational patterns support the use of the ARC to test the context-specific hypotheses about the contribution of temperament to AR.

### Bivariate relations of temperament with adaptive responding in novel and routine context

Bivariate correlations of the 15 CBQ subscales with parent rated ARC in Routine and Novel contexts are seen in Table 3. All of the NA subscales correlated with ARC in the Novel context but none correlated with ARC in the routine contexts. Fear and Sadness were negatively associated with ARC, whereas Anger and Falling Reactivity were positively associated. Two of the E/S subscales that aligned with emotion, hence with reactivity, Shyness and Smiling, correlated with AR in Novel but not Routine contexts, the former negatively and the latter positively. With respect to the EC subscales, context specific the patterns were not consistent. Two subscales, Attentional Focusing and Low Intensity Pleasure did not correlate with AR in either context. Inhibitory Control correlated with AR in both Routine and Novel contexts, but the relation was higher in the routine context (*z* = 1.68, *p* < 0.05). Finally, Perceptual Sensitivity was associated with AR in the Novel but not in the Routine context, and relations differed significantly (*z* = 2.45, *p* < 0.01).

**Table 3:** Correlations between ARC subscales, CBQ subscales, and composites.

CBQ factor	CBQ subscale	AR routine	AR novel
Effortful control		0.394**	0.386**
	Attentional focus	−0.014	0.195
	Inhibitory	0.567**	0.372**
	Control*		
	Perceptual	0.028	0.376**
	Sensitivity		
	Low-Intensity	−0.171	0.157
	Pleasure		
Negative Affectivity		0.139	0.497**
	Anger	−0.054	0.592**
	Fear	0.142	−0.335*
	Discomfort	0.072	−0.113
	Sadness	0.084	−0.359**
	Falling reactivity	−0.058	0.551**
Surgency/Extraversion		0.095	. 0.120
	Activity Level	−0.032	0.181
	Shyness	0.023	−0.325**
	High Intensity Pleasure	−0.044	−0.071
	Smiling	0.106	0.264*
	Impulsivity	0.039	−0.015
	Approach	−0.032	−0.072
	ARC Routine	-	0.369***
ARC Subscales			
	ARC novel	0.369***	-

***p < 0.001, **p < 0.01, *p < 0.05.

*Note:* A Fischer’s *Z* Test demonstrated that the ARC correlates to Inhibitory Control were significantly different (*z* = 1.68, *p* < 0.05); ARC, Adaptive Responsiveness to Context Scale; CBQ, Child Behavior Questionnaire.

### Path analysis

To test the primary hypothesis, we conducted an exploratory path analysis, controlling for overlaps between the two contexts. Incorporating both Novel and Routine ARC in the model accounted for overlaps in responses between the two contexts, hence highlight context-specific and unique contributions of each predictor. The model included temperament composites as the predictor variables and adaptive responsiveness (AR) in Novel and Routine contexts as the outcome variables utilizing the software program MPLUS (Muthén and Muthén, 2017).

#### Variable selection

To reduce the number of variables in the path analyses, we were guided by the three-factor conceptualization of the subscales that has emerged from higher order factor analyses (Rothbart et al., 2001; Teglasi et al., 2015). Three of four predictors were composites of CBQ subscales that aligned with positive emotional reactivity (E/S), negative emotional reactivity (NA) and regulation (EC). The fourth predictor, Falling Reactivity, is a subscale that loads inversely on NA, but is distinct from the others in that it does not have a positive or negative valence. However, as an aspect of NA, we hypothesized that it would be particularly relevant to ARC in the Novel context.

Negative Affect (NA) Composite (*α* = 0.86) represents an individual’s susceptibility to experiencing negative emotions (Rothbart et al., 2001). This composite was calculated as the mean of items on four subscales that load on this factor: Fear, Sadness, Discomfort and Anger. In order to explore its potentially unique effects, we entered the Falling Reactivity subscale (*α* = 0.79) separately.

Extraversion/Surgency Composite (*α* = 0.68), sometimes described as measuring levels of exuberance or excitability (Rothbart et al., 2001), is captured in the following subscales: High Intensity Pleasure, Impulsivity, and Activity Level. E/S also incorporates aspects of positive emotional reactivity captured in two subscales: Approach and Smiling. The E/S score was calculated as the mean of items on these two subscales.

Effortful Control Composite (*α* = 0.78), comprising the self-regulatory component of temperament (Rothbart, 2007), was calculated as the mean of all the items on subscales that load on this factor: Attentional Focus, Inhibitory Control, Perceptual Sensitivity, and Low Intensity Pleasure.

**Figure 1:**
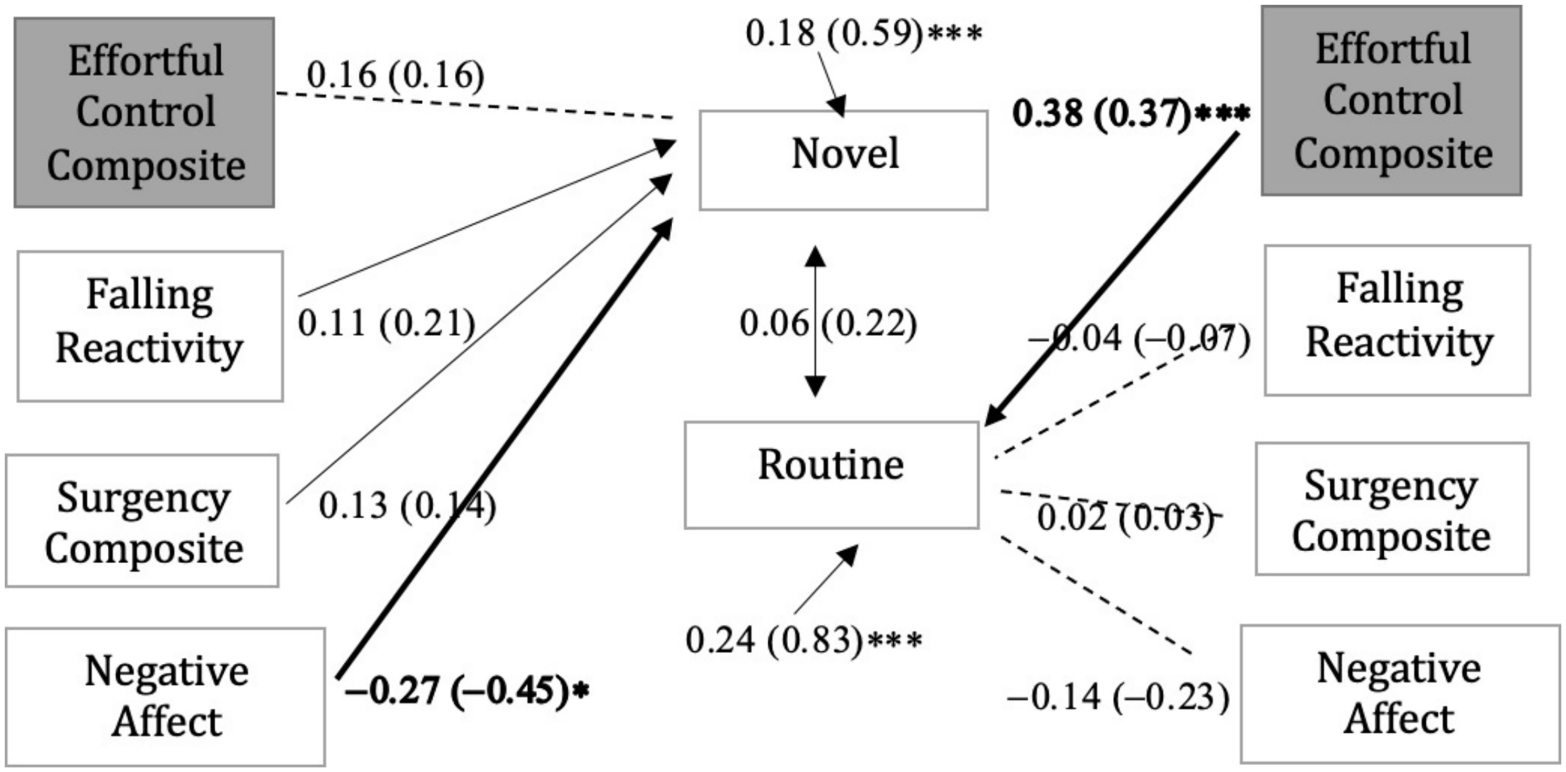
Structural model with traits measured by the Child Behavior Questionnaire. Each coefficient is followed by the standardized coefficient () in parentheses. The error variance indicates the amount of unexplained variance in Novel and Routine Adaptive Responsiveness. Solid lines indicate which relationships were hypothesized to be statistically significant, and bold arrows indicate the relationship was found to be statistically significant. No hypotheses were offered for relations indicated by the dotted lines. *p < 0.05, **p < 0.01, ***p < 0.001.

Results of the path analysis (see Figure 1) revealed that the Effortful Control Composite (*b* = 0.38, *β*=0.37, *p* < 0.001), was a unique (positive) predictor of Routine Adaptive Responsiveness and that the Negative Affectivity Composite (*b* = −0.27, *β*= − 0.45, *p* < 0.05) was a unique (negative) predictor of Novel Adaptive Responsiveness. As suggested by Kenny et al. (2015) fit indices are not reported for this model due to the small number of degrees of freedom (df = 0). In cases where degrees of freedom are small, fit indices can be misleading unless the sample size is large. The relations in the model explain 17 percent of the variance in Routine Adaptive Responsiveness (*R*^2^ = 0.17, *p* < 0.05) and 41 percent of the variance in Novel Adaptive Responsiveness (*R*^2^ = 0.41, *p* < 0.001). Overall, temperament as measured by parent reported CBQ is more predictive of functioning in novel than routine contexts.

## Discussion

A substantial body of work has shown that young children’s temperamental individuality is related to their social competence, but has not given systematic consideration to context. Yet, distinct patterns observed in children’s responses to novelty are explained by temperament theorists and researchers as a function of early appearing, reactive tendencies. In this study we re-examine previously established relations between temperament and social functioning in both Novel and Routine contexts. To pursue the aims of the study, we devised and validated a structured parent interview as a tool to measure young children’s adaptive responses in contexts (ARC) that are Novel and Routine. After establishing the validity of the ARC for the purposes of this study, we tested context-specific hypotheses about relations between preschoolers’ temperament and social functioning.

Feelings of uncertainty, evoked in contexts that are unfamiliar, ambiguous, or depart from expectations, are associated with negative emotional states, particularly fear/anxiety but also with other negative emotions, including being upset, angry, frustrated, sad or confused (see Morriss et al., 2022). Consistent with the emotion eliciting nature of Novel contexts, the temperament variables entered in the path model explained more variance in ARC in the Novel (41%) than the Routine context (17%). Moreover, parent rated ARC was higher in the Routine than in the Novel context, suggesting that a certain degree of wariness in new situations may be typical in a community sample of preschoolers. Although participants in this study do not represent extremes in temperamental reactivity, those with higher NA appear to be more vulnerable to uncertainties inherent in Novel contexts. As would be expected, there was some overlap in ARC across Novel and Routine contexts, evident in the moderate correlation (0.37) between them. Hence, we tested our primary hypothesis with an exploratory path model that controlled for variance shared across contexts and across temperamental dispositions.

Convincing evidence points to the adverse effect of negative affective reactivity (NA) and to the beneficial effect of effortful control (EC) on children’s social functioning using context-free methods (e.g., Eggum-Wilkens et al., 2016). However, relatively few studies have examined linkages between Extraversion/Surgency and social competence. Using path analysis, we revisited relations between temperament and adaptive social responses in Novel and Routine contexts. As anticipated, higher NA uniquely predicted lower ARC in the Novel context but did not contribute uniquely to ARC in the Routine context and, higher EC uniquely predicted higher ARC, in the Routine context but did not contribute uniquely to ARC in the Novel context. The finding that E/S did not emerge as a unique predictor of ARC was consistent with expectations based on the argument that various aspects and levels of E/S relate to social responses in different ways. Bivariate analyses provide insight into context-specific relations between ARC and individual scale subsumed within the composites.

### Bivariate analyses

#### Reactivity

Children’s initial responses to novel experiences range from *inhibited* (negative emotions; withdrawal) and linked to higher NA to *exuberant* (positive excitement, increased motoric activity, approach) and linked to higher E/S. These contrasting tendencies have been linked to physiological reactivity as well as to subsequent mental health outcomes (e.g., Dollar et al., 2017). In this study, whether the direction was positive or negative, all significant bivariate correlations between reactive traits (whether loading on NA or on E/S) and ARC were exclusive to the Novel context. With the exception of Anger, higher scores on negative emotions, were associated with lower ARC in the Novel context. This pattern is consistent with the finding of the path model wherein NA, in aggregate, contributed uniquely to ARC in the Novel but not Routine context. Although the E/S composite did not contribute uniquely to ARC in the Novel context, two of its subscales (Smiling and Shyness) did correlate with ARC in the Novel, but not in the Routine, context.

Higher scores on Anger, Falling reactivity and Smiling, all reactive aspects of temperament, were associated with higher ARC in the Novel context. The unexpected association of Anger with higher ARC in the Novel context may be explained in terms of a potential link with positive emotionality. Positive emotions in the face of novelty motivate children’s engagement (Stifter et al., 2008), which expands opportunities to gain social competence (Dollar et al., 2017). Again, in this study, the association of Smiling/Positive Emotions and of Anger with higher AR held only in the Novel context. Children with greater interest in novel experiences may be more likely to express anger/frustration when their approaches are limited by adults. Anger, as measured with the CBQ, taps into a child’s tendency to resist limits and to express frustration with thwarted goals. The functional perspective on emotions posits that each emotion motivates a particular goal and is associated with a certain way of thinking and acting (see Lench et al., 2015). Perhaps some degree of protest to adult limits (Anger/Frustration) by preschoolers is adaptive. As such, there may be important conceptual distinctions between certain emotions subsumed within NA, such as anger, sadness, and fear that are not well captured by the aggregate.

#### Effortful control

Children’s EC, thought to play a central role in self-regulation, is highlighted as key to children’s social–emotional development (e.g., Eisenberg et al., 2010; Rueda, 2012). With controls for context and trait overlaps, the composite EC score made a unique positive contribution to AR, but only in the Routine context. However, bivariate correlations between AR and the individual scales that make up this composite were not specific to the Routine context. Two of the four scales comprising EC, Inhibitory Control and Attentional Focus, have been far more extensively studied in relation to social functioning (e.g., Eisenberg et al., 2016) than the others, Perceptual Sensitivity and Low Intensity Pleasure. Perhaps the most understudied is Perceptual Sensitivity, which refers to the tendency to be receptive to low key stimuli and to notice subtle cues in the surroundings (Evans and Rothbart, 2007).

Inhibitory Control refers to children’s capacity to deliberately suppress a ‘ready’ response in favor of a more appropriate alternative, which requires the child to understand the difference. Other processes subsumed within the EC rubric, Attentional Focus, Low Intensity Pleasure, and Perceptual Sensitivity, refer to children’s capacity to process information that enables them to discern the need to suppress a ‘ready’ response. In this study, Attentional Focus and Low Intensity Pleasure did not correlate significantly with AR in either context. Inhibitory Control and Perceptual Sensitivity showed the expected positive correlations with AR but in different contexts. Inhibitory Control correlated with AR in both contexts, but more strongly in the Routine context, consistent with the path analysis. Among preschoolers, it is not the particular behavior, such as the expression of anger, that is troubling, but its inappropriateness to the context (Locke et al., 2015). The understandings that underlie effective exercise of Inhibitory Control are available in familiar contexts and, perhaps to a lesser degree, in novel contexts that include familiar elements.

Perceptual Sensitivity correlated with AR in the Novel but not the Routine context. Perceptual Sensitivity bears similarities to concepts described by others as having potential to play a role in social functioning: *novelty awareness*, (see Evans et al., 2012) and *sensitivity to subtleties* (see Aron and Aron, 1997). Children who are more aware of nuances would seem better positioned to detect social cues in novel contexts.

Overall, context played a key role in the link between temperament and social functioning. Eight of the 15 CBQ scales correlated with AR, but only one, Inhibitory Control, correlated in both contexts, but more strongly in Routine. Findings with respect to reactivity showed that, regardless of controls for context and trait overlaps, reactive traits are associated with AR in Novel but not Routine contexts. With respect to EC, when overlaps are controlled, the EC composite contributed uniquely to AR in the Routine but not Novel context. However, when disaggregated and tested without controls for overlaps, Perceptual Sensitivity correlated with AR in the Novel but not in the Routine context, whereas Inhibitory Control correlated with AR in both, but more strongly in the Routine context.

#### Limitations

This study has several shortcomings that may be addressed by future research. Specifically, the measure of AR in Novel and Routine contexts, though adequate to test the hypotheses, has not been extensively researched. In addition, although this study’s participants were ethnically/racially diverse, they were recruited from a single school.

Support for the hypothesized associations of the ARC with selective, theoretically relevant variables was taken as evidence for its valid use in this study. The Routine but not the Novel subscale correlated (positively) with conventional social skills and with conventional situation-emotion knowledge, whereas the Novel but not the Routine subscale correlated (inversely) with internalizing problems. Future efforts to devise context-specific questionnaires of children’s social functioning must grapple with complexities, including basic questions about how to conceptualize context. Our focus in this study was on context familiarity, given its relevance to temperamental reactivity. The essence of novelty is in not knowing what to expect, which elicits feelings of uncertainty that are magnified by NA. Temperamental reactivity is thought to be influential in the peer context because peer interactions involve constantly changing social cues and challenges that evoke emotions (Coplan and Bullock, 2012). Hence, even with familiar peers, interactions include some unknowns. Accordingly, the item on the ARC referring to “routine peer interactions” loaded on the Novel factor.

#### Implications for intervention

Children vary not only in their initial reactions to the uncertainties inherent to novel, changing, or unexpected conditions but also in how readily they acclimate, Perhaps it is the difficulty in making the transition from novelty to increasing familiarity, not the initial reactivity, that is problematic and should be targeted for intervention. For example, high reactivity in low threat contexts, reported by parents during toddlerhood, suggests difficulties acclimating, and is a more specific marker of risk for subsequent behavioral inhibition (see Buss et al., 2018).

Increasing familiarity with a context that once was new tends to reduce uncertainty as an elicitor of reactivity and may alter a child’s social functioning in that context. Moreover, gains in understanding that come with experience may enable the child to discern familiar elements in subsequently new encounters. For children with higher NA, this process of getting used to new experiences may take longer or may require more support. The challenge for interventionists is to unpack the processes that interfere with the child’s ability to gain a sense of predictability and agency with increasing familiarity.

The transactional view of child development provides an influential framework for early intervention/prevention programs for parents, caregivers, and educators of young children. Transactional models posit that development is shaped by the reciprocal dynamic between children and their surroundings and that temperament comprises the child’s contribution. Accordingly, the aims of temperament-informed interventions is to improve the match, or *goodness-of-fit*, between the adaptive demands of the context and the child’s temperament (Chess and Thomas, 1991).

From this transactional developmental perspective, strategies to improve fit would require unpacking the multiple interacting factors contributing to mismatches between the child’s behavior and others’ expectations in a given context. Consider a 5 year old who arrives with a parent at a busy and loud birthday party in a novel setting and immediately wants to leave, but the parent insists otherwise. Although this child gets along well with the pre-school peers attending the party, in this context the child is feeling overwhelmed. The parent, concerned with the child’s behavior, dismisses the child’s distress. For this child, the mismatch is the product of high reactivity, which makes it difficult to handle the adaptive requirements posed by (a) the *stimuli in the setting* and by (b) *others’ behavioral expectations.* Understanding the temperamental roots of the child’s behavior opens avenues for parents to consider both sources of mismatch.

Programs to promote children’s prosocial behaviors are often implemented as a vehicle to improve the fit between children and their peers. However, as in the above example, the child’s reason for wanting to leave the party had more to do with difficulty moderating reactivity in the setting than with the quality of peer relationships or of prosocial skills. In this vein, it has been suggested that social competence programs may benefit by considering the factors that underlie social behaviors, including variations in the motives, goals, and social strategies (see Kutnick et al., 2007; Garcia-Fernandez et al., 2022).

## Data availability statement

The raw data supporting the conclusions of this article will be made available by the authors, without undue reservation.

## Ethics statement

The studies involving human participants were reviewed and approved by Institutional Review Board University of Maryland. Written informed consent to participate in this study was provided by the participants’ legal guardian/next of kin.

## Author contributions

HV: conceptualization, data analysis, and initial writing. HT: conceptualization, development and validation of the ARC, methodology, editing, and re-writing. All authors contributed to the article and approved the submitted version.

## Conflict of interest

The authors declare that the research was conducted in the absence of any commercial or financial relationships that could be construed as a potential conflict of interest.

## Publisher’s note

All claims expressed in this article are solely those of the authors and do not necessarily represent those of their affiliated organizations, or those of the publisher, the editors and the reviewers. Any product that may be evaluated in this article, or claim that may be made by its manufacturer, is not guaranteed or endorsed by the publisher.
